# Neurosurgical Techniques for Disruption of the Blood–Brain Barrier for Glioblastoma Treatment

**DOI:** 10.3390/pharmaceutics7030175

**Published:** 2015-08-03

**Authors:** Analiz Rodriguez, Stephen B. Tatter, Waldemar Debinski

**Affiliations:** The Brain Tumor Center of Excellence, Department of Neurosurgery, Wake Forest University, Medical Center Boulevard, Winston Salem 27157, NC, USA; E-Mail: statter@wakehealth.edu

**Keywords:** blood–brain barrier, intra-arterial drug delivery, focused ultrasound, laser interstitial thermotherapy, non-thermal irreversible electroporation, glioblastoma

## Abstract

The blood–brain barrier remains a main hurdle to drug delivery to the brain. The prognosis of glioblastoma remains grim despite current multimodal medical management. We review neurosurgical technologies that disrupt the blood–brain barrier (BBB). We will review superselective intra-arterial mannitol infusion, focused ultrasound, laser interstitial thermotherapy, and non-thermal irreversible electroporation (NTIRE). These technologies can lead to transient BBB and blood–brain tumor barrier disruption and allow for the potential of more effective local drug delivery. Animal studies and preliminary clinical trials show promise for achieving this goal.

## 1. Introduction

**Figure 1:**
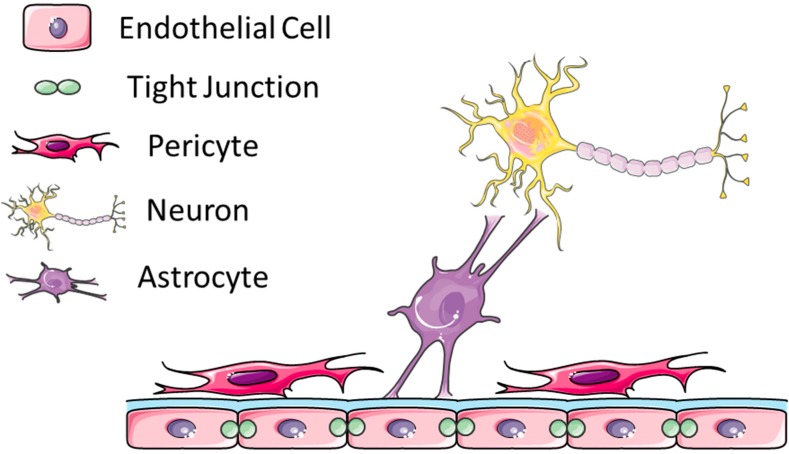
The blood–brain barrier is comprised of neurovascular units. Endothelial cells are connected by tight junctions and share a basement lamina with pericytes. Astrocytic end-feet are also at the basement lamina interface and these cells interact with neurons (This figure was developed using Servier Medical Art (http://www.servier.com/Powerpoint-image-bank) under a Creative Commons attribution 3.0 Unported License).

The blood–brain barrier (BBB) is critical for providing homeostasis and preventing biological toxins from entering the central nervous system. The main structural component of the BBB is comprised of non-fenestrated brain capillary endothelial cells that contain tight junctions. These endothelial cells are supported by pericytes and astrocytes which aid in stabilizing vessel walls and directing vessel development, respectively [1,2]. Pericytes and endothelial cells share a basement lamina which is contiguous with the plasma membranes of astrocytic end-feet. Astrocytes in turn communicate with neurons in order to allow for communication between brain vasculature and neuronal metabolic demand. This highly organized structural unit of the BBB is known as the neurovascular unit (Figure 1) [3].

Transport across the BBB can be via transcytosis, carrier-mediated transport, receptor-mediated transport, active efflux, or passive diffusion [4]. The BBB limits the diffusion of chemotherapeutics via the presence of endothelial tight junctions and low endocytic activity [5]. The selectivity of the BBB is based on lipid solubility and molecular size and charge. High lipophilicity and low molecular weight are properties that favor improved transfer across the BBB. The BBB does not allow for the diffusion of large and hydrophilic molecules. Therefore, lipid-soluble drugs with <400 Da weight are able to cross the BBB. Drugs that bind with serum proteins, such as albumin, are not able to cross the BBB, given their increased size.

Glioblastoma (GBM) remains the most common primary brain tumor in adults and poses a great therapeutic challenge with a median survival of only 15 months after diagnosis [6]. Glioma tumor cells undergo many biological changes that allow them to invade the surrounding normal brain. This allows for the tumor to evade treatment by infiltrating the surrounding area [7]. This migration of glioblastoma cells within the brain makes complete resection of this tumor impossible in the vast majority of cases. While some tumor cells have migrated to the contralateral hemisphere at the time of tumor presentation, the majority of tumor cells have migrated only several centimeters from the enhancing tumor apparent on neuroimaging scans [8]. Most tumors will recur within 4 cm of the original lesion, making local control an important factor in determining overall survival. Therefore, the development of local therapeutic delivery modalities is clinically useful, but many of the chemotherapeutics that have killed glioma cells in culture are not able to cross the BBB and therefore do not have significant *in vivo* therapeutic effects [9].

Brain tumors are capable of disrupting the connection between astrocytes and brain endothelial cells, which can destabilize the BBB [10]. In GBM, glioma cancer cells invade along pre-existing blood vessels and are able to displace the astrocytic end-feet from endothelial cells [11]. Endothelial tight junctions are impaired in glioma because of under-expression of tight junction proteins such as occludin, and over-expression of aquaporin-4 [12,13]. The BBB is heterogeneous within different regions of the tumor tissue. Usually the most permeability is found in regions where normal tissue has been replaced entirely by neoplastic cells, however, there is often an intact BBB at the border of the tumor where glioblastoma cells infiltrate into normal brain parenchyma [8]. Therefore, increasing permeability of the BBB at the tumor border has implications for improving therapeutics and, ultimately, patient outcome.

The BBB is often targeted by clinicians directly or indirectly and is a secondary effect of several medical treatments. Most notably, corticosteroids are routinely used for management of tumor-associated cerebral edema. Corticosteroids reduce edema by decreasing the permeability of tumor capillaries, upregulating tight junctions, and modulating vascular endothelial growth factor (VEGF) expression [14]. Anti-VEGF-A monoclonal antibody (bevacizumab) is used as an anti-angiogenic therapy with known effects on the BBB by normalization of abnormal tumor vasculature leading to reduced permeability [15]. In contrast, radiation, which is a standard adjuvant therapy to surgery, can lead to the increased permeability of the BBB [16]. In clinical practice the extent and timing of the disruption of the blood brain barrier by radiation is unpredictable and most often manifests as a complication rather than a therapeutic opportunity.

Several invasive and non-invasive neurosurgical approaches exist that allow for temporary disruption of the BBB and, therefore, the potential administration of therapeutic agents to the brain. Local disruption of the BBB and subsequent therapeutic delivery has the potential to affect recurrence and, in turn, overall survival. We herein review superselective intra-arterial mannitol infusion (hyperosmotic therapy), focused ultrasound (FUS), laser interstitial thermotherapy (LITT), and non-thermal irreversible electroporation (NTIRE). Hyperosmotic therapy was developed decades ago but is not routinely used in the clinical setting due to its many limitations. Technological advances in focused ultrasound have led to this method becoming more popular. LITT and NTIRE are also known to lead to the disruption of the BBB but do not yet even have preclinical studies that investigate the potential for delivering chemo-therapeutics following treatment. Nonetheless, these techniques hold promise for improving management of GBM in the future.

## 2. Superselective Intra-Arterial Mannitol Infusion

**Figure 2:**
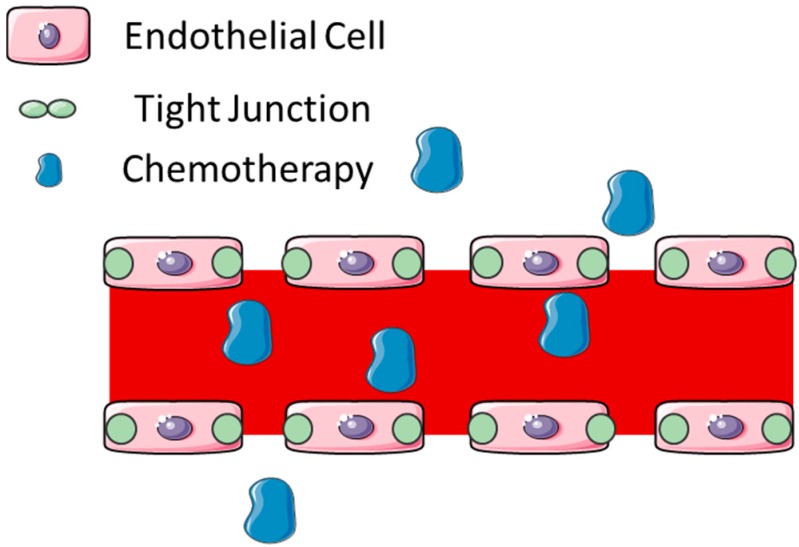
Schematic of mechanism of blood–brain barrier disruption by intra-arterial mannitol administration. The hyper-osmolar agent causes endothelial cell dehydration and subsequent shrinkage as well as tight junction disruption. This allows for increased permeability across the blood–brain barrier (This figure was developed using Servier Medical Art (http://www.servier.com/Powerpoint-image-bank) under a Creative Commons attribution 3.0 Unported License).

Blood–brain barrier disruption (BBBD) via intra-arterial (IA) administration of osmotic agents was first used in patients in the 1970s. Prior to administration of a drug, the BBB is opened using an osmotic agent, most commonly mannitol, which has been utilized for this purpose in preclinical and clinical trials. Endovascular access to the tumor is required and a catheter is placed in the main feeding artery of the tumor. A standard dose of 10 mL of 1.4 M mannitol is infused over two minutes, followed by the chemotherapeutic agent of choice [17]. Injection of an osmotic agent causes shrinkage of the endothelial cells and the subsequent opening of the tight junctions (Figure 2) [18]. The use of an osmotic agent is estimated to increase drug delivery by 10 to 100 times in comparison to delivering the drug alone [19]. The barrier remains open for up to 2 to 3 h [20].

IA drug delivery is theoretically most effective in the presence of low regional blood flow, high regional extraction, and high systemic clearance. Uneven distribution of the drug can lead to regions of the brain receiving high concentrations that can lead to the development of neurological deficits. As endovascular techniques advanced, it became possible to control blood flow and inject boluses of drugs that can direct drugs to specific sites [21]. The ideal drug used in IA delivery for the treatment of brain tumors should be highly extracted during its first pass, have an increased permeability surface area product that can be improved by decreasing polar groups or increasing aliphatic groups, and have a very short half-life so that the drug is metabolized while in the location of the tumor [21]. Liposomal formulations have been developed in order to improve first-pass extraction in brain tissue. Cationic carriers have also shown promise in drug delivery [8].

Following mannitol-based disruption of BBB, multiple agents have also been administered intra-arterially, such as carmustine, cyclophosphamide, procarbazine, methotrexate, and doxorubicin. For patients with GBM, the intra-arterial cerebral infusion of bevacizumab, cetuximab, and temozolomide has been examined, but there are no randomized trials demonstrating clinical benefit in patients with GBM [22]. Despite this long history, hyperosmotic blood–brain barrier disruption is not part of standard practice in part because it requires repeated hospitalizations, often necessitates general anesthesia, and it increases the risk of seizures and strokes. In addition, tumors do not adhere to specific vascular distributions, so the targeting of multiple vessels may be needed for therapeutic efficacy.

Preliminary clinical trials of the administration of IA bevacizumab (BV) for recurrent GBM have demonstrated that it is safe and can lead to reduced enhancing tumor volume [23]. Another study of these patients demonstrated that IA BV can increase progression-free and overall survival in comparison to historical controls [17]. This study was completed in only 14 patients but nonetheless demonstrates the promise of this technique. IA BV can also be administered to areas of the brain that are not amenable to surgical resection, such as the brain stem [24].

Despite these promising preliminary clinical trial results, the main issue that currently limits using IA delivery for glioma is pharmacokinetics because chemotherapeutics were not specifically designed for IA delivery. Also, there are potential risks of mannitol infiltration, including altered glucose uptake, the passage of plasma proteins across the BBB, and microembolism in the cerebral vasculature [8]. The lack of specific targeting is another limitation of IA therapy. To better localize therapy, some researchers have used transient flow arrest, which has been demonstrated to be safe and efficacious in the treatment of retinoblastoma [25,26]. However, endovascular flow arrest carries the risk of stroke.

## 3. Focused Ultrasound

**Figure 3:**
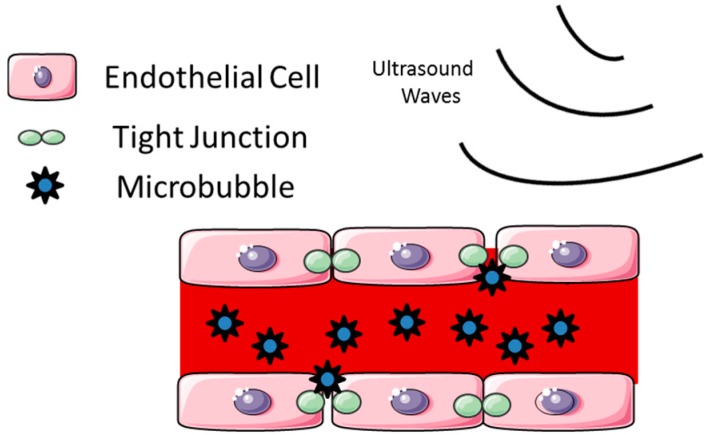
Schematic of blood–brain barrier disruption by focused ultrasound. Disruption of the blood–brain barrier can be induced when microbubbles apply mechanical forces on endothelial cells that lead to openings of the tight junctions (This figure was developed using Servier Medical Art (http://www.servier.com/Powerpoint-image-bank) under a Creative Commons attribution 3.0 Unported License).

Focused ultrasound (FUS) is a noninvasive technique in which low frequency ultrasound waves are delivered transcranially. Low frequency delivery is preferred in order to minimize permanent tissue damage and reduce absorption in the skull [27]. FUS can be used to thermally ablate tissue. The addition of microbubbles to FUS can also enhance the local heating in the area of focus [28,29]. However, at lower exposures, FUS with microbubbles can be used for BBBD. Ultrasonic exposure bursts at 10 ms repeated at the frequency of 1 Hz used for 20–30 s durations are the typical settings used. Clinically, FUS is conducted in conjunction with magnetic resonance imaging (MRI) to confirm the region of interest and to assess the area of BBBD and/or thermal ablation. Focused ultrasound with the use of microbubbles can induce local and reversible BBBD by altering tight junctions in the cerebrovasculature. When the microbubbles interact with even low-intensity ultrasound, mechanical forces on the endothelium can cause transient opening of the tight junctions (Figure 3). These microbubbles are delivered intravenously and are composed of lipid-encased perfluorocarbon gas approximately 1–5 µm in diameter [30,31]. To manipulate the area of BBBD, the size and resonance frequency of the microbubbles can be altered. With larger microbubbles, less acoustic pressure is needed to achieve BBB opening. With focused ultrasound the BBB remains open for several hours and can be localized to the tumor region. The BBBD is transient and reversible [32]. The size of BBBD in FUS can be controlled by acoustic pressure, allowing for agents up to 2000 kDa to enter. Thus, the size selectivity of BBB disruption can be controlled by FUS [33]. Dynamic contrast-enhanced MRI has been shown to be able to monitor the kinetics of BBBD by FUS [34].

Preclinical studies have been conducted to assess the feasibility of BBBD by FUS for the administration of chemotherapeutics in the treatment of glioma. In a rat glioma model, survival was increased in animals that received FUS in combination with liposomal doxorubicin [35]. In yet another preclinical study, FUS increased the concentration of temozolomide in the brain and this correlated with decreased tumor progression and increased animal survival [36]. High intensity FUS has also been successfully used to deliver receptor-targeted liposomes to brain tumors in a mouse model [37]. This method of BBBD can also be used to deliver nanoparticles, DNA, plasmid vectors, and antibodies [38,39,40,41].

Initially, many barriers existed prior to making FUS clinically feasible. Ultrasound beams could be noninvasively focused in the brain through the skull in patients, but there was insufficient power to achieve ablation. Another obstacle encountered in the study was the lack of successful real-time MRI monitoring of the entire brain volume at risk of thermal injury [42]. In the last several years, these barriers have been overcome and FUS is beginning to be used for brain tumors. One case study has been reported in which FUS was used to treat a patient with recurrent GBM. To achieve ablation of the tumor, 25 sonications of 10–25 s duration and 150–950 Watts of acoustic power were applied. Several-minute waiting periods between sonications are required to allow cooling of the skull. After five hours, 0.7 cc of the tumor was ablated, which was 10% of the enhancing tumor volume [43]. Therefore, since FUS for tumor ablation can now be done clinically, BBBD by transcranial-focused ultrasound with microbubbles in patients seems feasible in the near future and is ripe for studies optimizing its use.

## 4. Laser Interstitial Thermotherapy

Laser interstitial thermotherapy (LITT) is a novel technique that allows for laser ablation of a tumor via insertion of an optical fiber. LITT is minimally invasive and, like FUS, destroys tissue through the administration of heat. The laser-generated heat induces necrosis of the tumor. LITT has been used in many tissue types, such as liver, lung, and prostate [44,45]. In the brain, laser thermotherapy has been reported for the treatment of glioma, metastases, and radiation necrosis [46,47,48,49,50,51]. LITT can also be used to treat lesions in locations that are not amenable to standard open surgery [52]. In a prospective trial on the use of LITT for recurrent glioma, there was a trend toward improved survival in patients treated with higher thermal doses [47]. Progression-free survival was also improved in patients with high-grade glioma in difficult-to-access areas when there was more complete coverage of tumor volume by thermal ablation treatment lines [53]. These results imply that enhanced thermal ablation likely portends survival benefits to patients analogous to the effect of the increased extent of glioma resection on patient survival.

To perform LITT, a cooled YAG laser is placed stereotactically in the lesion and used to provide thermal energy to ablate the lesion. LITT is monitored with real-time magnetic resonance imaging (MRI) thermometry and software is available to sum the regions heated sufficiently to achieve thermal ablation [47]. Thermotherapy can induce cell membrane destruction and ultimately results in coagulative necrosis. Cell membrane destruction in endothelial cells can cause disruption of the BBB and allow for the passage of chemotherapeutic drugs [54]. Gadolinium-infused MRI following LITT shows an area of enhancement around the thermal ablation zone. This area of enhancement is the result of increased blood–brain barrier permeability [55]. In a non-human primate model, laser ablative hyperthermia induced BBBD for up to several days following treatment [56]. While LITT is used clinically, no studies have yet investigated whether chemotherapeutic delivery is enhanced during the time interval that the BBB remains open following LITT treatment.

## 5. Non-Thermal Irreversible Electroporation

**Figure 4:**
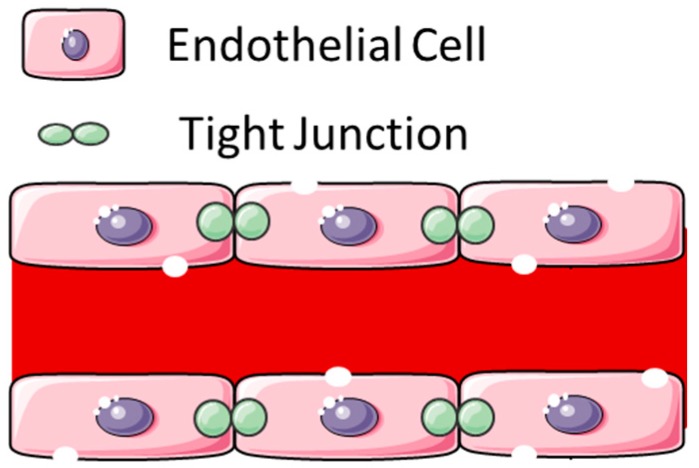
Schematic of blood–brain barrier disruption by electroporation. A pulsed electric current causes direct defects into the cell membrane of the endothelial cells, resulting in increased permeability (This figure was developed using Servier Medical Art (http://www.servier.com/Powerpoint-image-bank) under a Creative Commons attribution 3.0 Unported License).

Another modality of tissue ablation that also results in blood–brain barrier disruption is non-thermal irreversible electroporation (NTIRE). NTIRE uses a pulsed electric current to increase permeability of the cell membrane by causing defects in the cell membrane and, ultimately, membrane rupture (Figure 4) [57]. Electrodes are placed stereotactically in the brain after a standard cranial opening and an electric current is applied. The effects of the electric current only affect the cell membrane and not the extracellular scaffold. This has been advantageous as structures such as blood vessels can remain undamaged and continued surrounding tissue regeneration can occur [58]. In the brain, NTIRE-applied voltage correlates with both the volume of tissue damage and BBBD [59]. In rat models, the BBBD can last several days post-treatment [60]. Animal models of NTIRE demonstrate that an area of BBBD is present surrounding the area of IRE-induced cell death across ranges of electric fields [61,62]. NTIRE has been used to ablate a glioma in a canine and was successful in reducing the tumor volume [63]. NTIRE has not yet been reported in patients for the treatment of glioma or in combination with chemotherapy. Clinical feasibility trials are needed to assess for safety.

## 6. Conclusions

The four methods for BBBD for the delivery of chemotherapeutic agents for glioma each have advantages and disadvantages. Both IA infusion and FUS lead to the disruption of the BBB for several hours, whereas LITT and NTIRE produce disruptions that may last for several days. Thus far, only IA therapy has been reported clinically in glioma patients for the delivery of chemotherapeutics after BBBD, but has not led to significant improvement in patient outcomes. FUS is the only non-invasive method of BBBD and has begun to be used clinically for tumor ablation. However, this method has not been reported to treat a significant tumor volume in the human brain. LITT is a neurosurgical intervention that is in use clinically, but its potential as a method to deliver therapeutic agents after BBBD has yet to be exploited. Currently, LITT is solely used to thermally ablate lesions. Like LITT, NTIRE also has the potential to both destroy the tumor cells directly and allow for a perimeter of BBBD. This may be of great value, especially in the face of glioma treatment, as the perimeter surrounding the tumor is the volume most prone to disease recurrence. As these methods are optimized to be used in the clinic, the ideal agents to use and the timing of delivery will need to be determined.

Disruption of the BBB remains a promising concept to deliver therapeutic agents to the brain. Glioblastoma remains one of the most difficult-to-treat cancers given the lack of penetration by potentially potent drugs. Even once the BBB is disrupted, the clinician must decide what the best agent to deliver is. Novel methods are in development to improve drug delivery, such as the use of nanoparticles. The engineering of nanoparticles can enhance a drug’s permeability across the BBB [64,65]. The delivery of monoclonal antibodies, viral vectors, and stem cells is also on the horizon [66]. Besides improving and combining existing methods of drug delivery, recent discoveries on the major regulators of BBB development may hold the key to developing novel therapeutics [67]. Three-dimensional culture techniques are being developed to improve *in vitro* models of the BBB in order to test novel therapeutics [68,69]. New imaging techniques such as fluorescence imaging and contrast-enhanced nuclear imaging have the potential to quantitatively assess cellular processes of the BBB [70]. Continued innovation is needed to advance the current methods of BBBD to clinical applications in patients.
